# Predicting Temporal Liking of Food Pairings from Temporal Dominance of Sensations Data via Reservoir Computing on Crackers and Spreads

**DOI:** 10.3390/foods14193373

**Published:** 2025-09-29

**Authors:** Hiroharu Natsume, Shogo Okamoto

**Affiliations:** Department of Computer Science, Tokyo Metropolitan University, Hino 191-0065, Japan; natsume-hiroharu@ed.tmu.ac.jp

**Keywords:** food combination, reservoir computing, cracker, spread

## Abstract

The temporal dominance of sensations (TDS) and temporal liking (TL) methods offer complementary insights into the evolution of sensory and hedonic responses during food consumption. This study investigates the feasibility of predicting TL curves for food pairings from their TDS profiles using reservoir computing, a type of recurrent neural network. Participants evaluated eight samples—two crackers (plain, sesame), two spreads (peanut butter, strawberry jam), and their four binary combinations—performing both TDS and TL evaluations. This process yielded paired time-series data of TDS and TL curves. We trained various reservoir models under different conditions, including varying reservoir sizes (64, 128, 192, or 256 neurons) and the inclusion of auxiliary input dimensions, such as flags indicating the types of foods tasted. Our results show that models with minimal auxiliary inputs achieved the lowest root mean squared errors (RMSEs), with the best performance being an RMSE of 0.44 points on a 9-point liking scale between the observed and predicted TL curves. The ability to predict TL curves for food pairings holds some promise for reducing the need for extensive sensory evaluation, especially when a large number of food combinations are targeted.

## 1. Introduction

The temporal dominance of sensations (TDS) [1,2,3] and temporal liking (TL) [4,5] methods allow recording of sensory and hedonic dynamics during food consumption. By combining the temporal evolution of dominant sensations captured by the TDS method with the numerical hedonic evaluations provided by the TL method, researchers have attempted to elucidate the temporal relationship between perception and liking [5,6,7,8,9,10,11,12,13,14,15,16,17,18,19]. These approaches aim to identify which sensory attributes—such as taste, aroma, and texture—determine liking at each moment or interval during consumption. For example, a pioneering study proposed using the interval mean of liking scores during periods when a specific attribute is dominant as an indicator of the sensory drivers of liking [5]. This approach was later refined by weighting each attribute according to its dominance duration [11]. Kuesten and Bi [16] repeatedly conducted time intensity methods for different sensory attributes instead of the TDS method, and investigated how those intensities related to the temporal liking of fruit chews. They used a non-additive model to connect the sensory and liking data, and then employed relative importance measures used in machine learning and statistic learning techniques as evolving temporal drivers of liking. Okamoto et al. demonstrated the validity of vector autoregression [17] and state-space [18] models that determine the current liking value based on the current and past sensory properties of food items. Long before the advent of the TDS method, numerous studies had investigated the relationship between sensory attributes such as taste, aroma, and texture and consumer liking. Classic review articles and textbook chapters (e.g., [20,21,22,23,24]) comprehensively summarize analytical approaches such as preference mapping, regression, and multivariate analysis for linking sensory profiles to hedonic responses.

In connection with these techniques, our group has developed methods for predicting TL outcomes based on TDS data. For example, we have used neural networks to estimate corresponding TL curves from TDS curves for strawberries and coffee [8,25]. A primary motivation for this work is to reduce the cost of sensory evaluation or consumer tests and to streamline the food development process—a goal widely shared among food industry practitioners [26,27,28]. Sensory evaluation is employed in nearly 70% of food development cases, involving both expert panels and consumers, and can represent a major cost driver in the process [29]. Consequently, the development and adoption of rapid sensory profiling methods, consumer-oriented approaches, and predictive modeling, including machine learning approaches, have become important topics for reducing the time and expense of sensory-driven innovation.

A scenario in which cost efficiency becomes particularly important is the evaluation of food pairings, which have long been of great interest in sensory science [30,31,32,33]. For example, when considering combinations such as drinks and foods, even with only 10 varieties of each, the number of possible pairings reaches 100. Conducting sensory tests on all these combinations would substantially increase costs. Therefore, there is value in developing methods that can identify favorable food pairings while minimizing the cost of sensory evaluation. To this end, for example, Kustos et al. [33] efficiently investigated compatible food and wine pairings by combining descriptive sensory analysis with hedonic evaluation.

Several research groups have investigated methods for food pairing using machine learning [34]. For example, Ahn et al. [35] analyzed a global recipe database to identify preferred ingredient combinations. Similarly, Park et al. [36] estimated the compatibility of ingredient combinations using neural networks trained on recipe data. Furthermore, Park et al. [37] developed a method based on a knowledge graph to assess ingredient pairing compatibility. These approaches aim to discover favorable ingredient pairs from large-scale databases. In contrast, our study evaluates the quality of ingredient pairings by applying machine learning to TDS and TL data—a fundamentally different approach. To our knowledge, there have been no prior studies that have used TDS and TL methods to estimate the quality of ingredient pairings. While Galmarini et al. [19] used TDS and TL methods to investigate the temporal drivers of liking for wine and cheese pairings, their objective was not to predict pairing compatibility. Considering the growing popularity of TDS methods in food science and their adoption in industry [38], developing approaches that can predict liking from TDS data could provide an effective tool for supporting product innovation and reducing the cost of sensory testing in the food industry.

**Figure 1:**
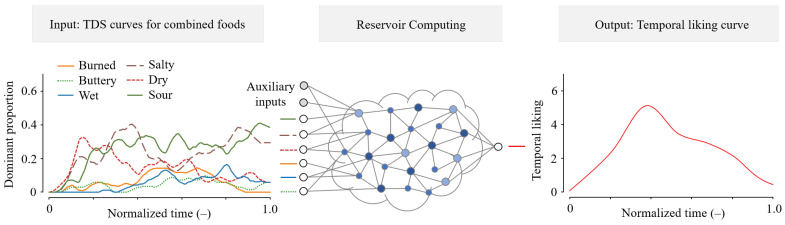
Estimation of a TL curve from TDS curves obtained when two foods are tasted in combination.

In this study, we develop a method for estimating the compatibility of food pairings using TDS and TL curves. As a prediction method, we employ reservoir computing [39,40,41], a type of recurrent neural network well suited for handling time-series data. We focus on two types of crackers and two types of spreads. TDS and TL curves are collected for each item when tasted alone and in combination. As shown in Figure 1, the reservoir computing model trained on these data predicts the TL curve for a given food pairing based on its TDS curve. Although predicting both the TDS and TL curves for pairings directly from the TDS curves of individual items remains an aspirational research direction, this study represents an initial attempt to explore feasibility within a limited context.

## 2. Methods: Sensory Evaluation and Data Processing

### 2.1. Food Samples

We used two types of crackers (plain, sesame) and two types of spreads (peanut, strawberry). The plain crackers were Premium Saltine Crackers (Nabisco, Inc., East Hanover, NJ, USA; lot number: 24 OCA00433D2). The sesame crackers were Black Sesame Soft Crackers (Maeda Confectionery Co., Ltd., Sakai, Osaka, Japan; best-before date: 14 October 2025). The peanut butter was Verde Peanut Whip (Aohata Corporation, Takehara, Hiroshima, Japan; production number: 2026.3/DA2; best-before date: March 2026). The strawberry jam was Spoon Free Strawberry (Aohata Corporation, Takehara, Hiroshima, Japan; production number: 2026.3.4/C; best before date: March 2026). The names and manufacturers of the individual products are listed in Table 1. These foods were selected due to their ease of handling, consistent quality and availability, and suitability for combination consumption.

Crackers were served as bite-sized pieces, and spreads were served using a spoon (3.5 g). In addition to these individual items, we prepared four cracker–spread combinations: plain–peanut, plain–strawberry, sesame–peanut, and sesame–strawberry. In the combination task, bite-sized crackers were served with 3.5 g of spread applied on top.

In this study, two types of crackers and two types of spreads were selected, resulting in four pairings and, together with the single-item conditions, eight tasting conditions in total. This number of samples was selected to balance the variety of foods examined with the practical feasibility of the experiment (see Section 2.3).

### 2.2. Panel

Sixteen university students (age: 20–25; 5 females and 11 males) participated as the panel in the experiment. Prior to the experiment, participants confirmed that at least two hours had passed since their last meal and that they did not dislike the food being served. While this panel size satisfies the minimum recommendation for semi-trained panels specified in ISO 13299 [2], the homogeneity of the panel in terms of age and background is a limitation that should be considered when generalizing the findings.

The panel received training focused on the identification of sensory attributes. Participants first reviewed and agreed upon definitions of the TDS attributes. Two training sessions were conducted to familiarize them with the TDS method, including practice in selecting dominant sensations and becoming accustomed to the computer interface used for data collection. During these sessions, participants practiced with sample foods not included in the main experiment. In addition, all participants were familiarized with the TL task and the use of the nine-point liking scale. They completed two practice sessions in which they provided real-time liking responses to sample foods using the interface.

### 2.3. TDS and TL Tasks

The TDS and TL experiments were conducted in a quiet room maintained at approximately 23 °C. Each participant evaluated two types of crackers, two types of spreads, and all four possible combinations of these foods. After tasting each food sample, participants rinsed their mouths with soft water. The order of food sample presentation was randomized, as was the sequence of TDS and TL tasks for each food. The TDS and TL tasks were conducted separately, with each task repeated three times per food item. In total, each participant completed 48 trials (8 food conditions × 2 tasks × 3 repetitions), which required approximately two and a half hours.

We used custom-made software written in Python 3 (version 3.13.3) to collect the TDS and TL data. For the TDS task, participants assessed changes in their dominant sensations using a graphical interface with buttons labeled by sensory attributes. At any moment, participants pressed the button corresponding to the attribute they perceived as most dominant, switching buttons when their perception changed. Only one attribute could be selected at a time.

Twelve sensory attributes were used in the TDS tasks: aromatic, burned, buttery, dry, nutty, salty, sesame-like, smooth, sour, sweet, wet, and wheat-like. These attributes were selected by consensus among the authors and their three colleagues from a list compiled via web sources and other references [42,43,44,45,46,47]. The authors and three colleagues independently evaluated this list using a check-all-that-apply procedure to judge which attributes were applicable to the food samples. Attributes selected by at least four of the five evaluators were retained, yielding the final set of twelve. The same set of attributes was used for crackers, spreads, and their combinations. Definitions of these attributes are provided in Table 2.

For the TL task, participants also used a graphical interface with nine buttons labeled 1 through 9. While tasting each food, participants indicated their liking in real time by pressing the button that best represented their current preference—selecting 9 for most favorable and 1 for least favorable.

### 2.4. Dataset Generation with Bootstrap Resampling

The TDS task yielded TDS curves representing the temporal changes in dominance proportion for each sensory attribute. The dominance proportion for an attribute is defined as the proportion of trials in which that attribute was selected at each time point.

Similarly, the TL method produced TL curves, which show the temporal changes in the average liking score. Although the minimum possible score in the experiment was 1, the score was initialized to 0 at the beginning of each trial and remained 0 until the first button was pressed.

The TDS and TL tasks provide average time-series curves that represent the collective experiences of all participants. However, this results in a dataset that is insufficient for training machine learning or statistical models. To address this, we applied bootstrap resampling—a data augmentation technique—to the TDS and TL curves [48,49]. Initially, there were 16 pairs of TDS and TL curves, one pair for each participant. Bootstrap resampling was performed by randomly sampling 16 pairs with replacement from the original set of 16 panels, then averaging the sampled data to produce a single pair of TDS and TL curves. By repeating this process, we generated 100 curve pairs for each of the eight food samples.

## 3. Prediction of TL Curves Based on TDS Curves

### 3.1. Reservoir Model Architecture

To predict TL curves, we employed a reservoir model [39,40,41], a type of recurrent neural network well suited for time-series data. The model outputs a TL curve when provided with TDS curves as input. For the implementation, ReservoirPy [50] (version 0.3.12), a Python library for reservoir computing, was used.

The architecture consisted of three layers: input, reservoir, and output. The input layer comprised 12 nodes, each corresponding to a sensory attribute. The output layer was a single node, representing the scalar liking score. The number of reservoir neurons was varied across four configurations: 64, 128, 192, and 256. Based on previous studies [8,25], we considered it reasonable to expect that the optimal number would fall within this range.

In addition to the 12-dimensional attribute input, we explored seven alternative configurations that incorporated auxiliary information. These auxiliary inputs were encoded as flag vectors, where each dimension was set to 1 if the corresponding condition was satisfied and 0 otherwise. The purpose of the auxiliary input flags was to provide contextual information—such as whether the sensory evaluation was for a cracker or spread alone, or a combination—in an effort to improve prediction performance. We compared various architectures, differing in both the number of auxiliary input nodes and the number of reservoir neurons, with respect to prediction accuracy.

Table 3 summarizes the total input dimensions, the number of auxiliary dimensions added, and provides a description of each configuration.

**Figure 2:**
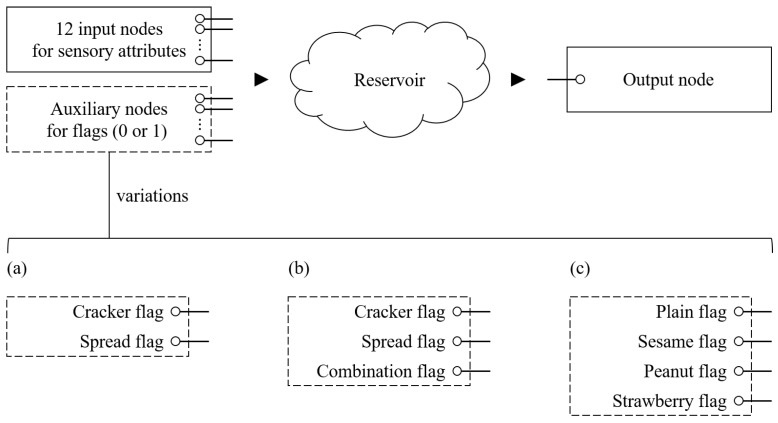
Structure of the input layer. (**a**) Two auxiliary nodes. (**b**) Three auxiliary nodes. (**c**) Four auxiliary nodes.

For example, as illustrated in Figure 2a, when cracker and spread flags were included, the input layer consisted of 14 nodes: 12 representing sensory attributes and 2 representing the flags. For single foods, only the corresponding flag was set to 1, while for paired samples, both flags were set to 1. For example, during the training phase, when the TDS curves for crackers were provided to the model, the cracker flag was set to 1 and the spread flag was set to 0.

As shown in Figure 2b, when cracker, spread, and combination flags were used, the input layer comprised 15 nodes. For paired foods, only the combination flag was set to 1, with the others set to 0.

In Figure 2c, when four brand flags were used, each flag indicated the presence of a specific ingredient (e.g., premium cracker, sesame, peanut, or strawberry). For instance, for a sample of premium cracker with strawberry jam, the premium and strawberry flags were set to 1, while sesame and peanut flags remained 0.

### 3.2. Training Dataset

The training data included TDS and TL curves collected from both single food items and pairings. The dataset for pairings was used in a cross-validation framework. For example, when predicting the TL curve for the plain–strawberry combination, the model was trained on the other seven food samples: plain, sesame, peanut, strawberry, plain–peanut, sesame–peanut, and sesame–strawberry. There were 100 resampled curve sets for each tasting condition; hence, each model was trained on a total of 700 curve sets.

### 3.3. Model Evaluation Metrics

The trained models were evaluated using a set of 100 curve pairs that were not included in the training data. For each curve set, the model predicted the TL curve by inputting the corresponding TDS curves, and the root mean squared error (RMSE) between the predicted and actual TL curves was calculated. This procedure was repeated for all 100 curve sets. Finally, the median and interquartile range (IQR) of the 100 RMSE values were calculated.

## 4. Results

**Figure 3:**
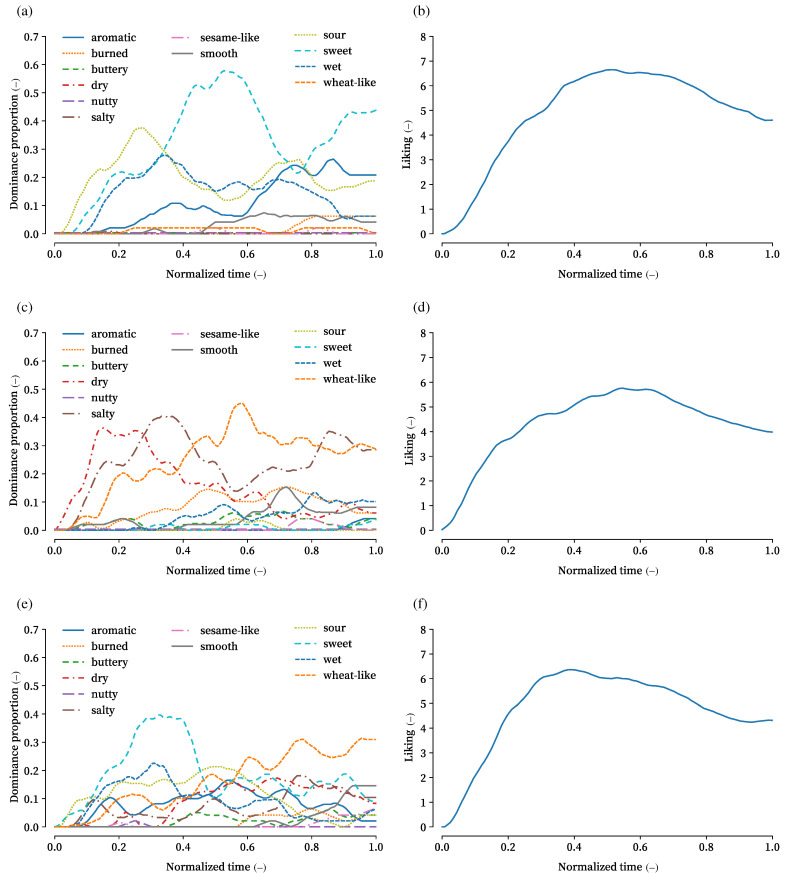
Examples of TDS and TL curves. (**a**) TDS curves of strawberry jam. (**b**) TL curve of strawberry jam. (**c**) TDS curves of plain cracker. (**d**) TL curve of plain cracker. (**e**) TDS curves of plain–strawberry. (**f**) TL curve of plain–strawberry.

As an example, Figure 3 shows the TDS and TL curves for strawberry jam, plain cracker, and their pairing. For strawberry jam in (a), “sour” initially appeared as the dominant attribute, followed by “wet”. In the middle phase, approximately 60% of panels selected “sweet”. The jam received its highest liking scores during this middle phase, exceeding 6, as shown in (b). For the plain cracker in (c), “dry”, “salty”, and “wheat-like” attributes were each perceived as dominant at different times. The TL curve in (d) resembles that of the strawberry jam, although its maximum value was below 6. For the combination of strawberry jam and plain cracker in (e) and (f), a wider range of attributes was selected than for the individual items. Attributes characteristic of the jam, such as “sour”, “wet”, and “sweet”, appeared more frequently in the early phase, while cracker-related attributes like “dry” and “wheat-like” appeared later. In the curves, “salty” and “dry” were selected less frequently than in the cracker-alone condition. In the TL curve, the peak liking score was reached earlier than for the individual items, around 0.4 on the normalized time scale, with the maximum score exceeding 6.

The observed TL curves can be interpreted in light of the sensory attributes identified in the TDS tasks. For example, plain crackers were predominantly characterized by dry, salty, and wheat-like attributes, which corresponded with lower liking scores that did not exceed six points. In contrast, strawberry jam was described as sour, sweet, and wet, attributes that generally align with higher hedonic responses, and indeed its TL curve reached higher values. When the two foods were paired, the TDS curves revealed a complementary pattern: sour, sweet, and wet attributes dominated early in the tasting, while dry and wheat-like attributes appeared later. This temporal interplay likely contributed to the higher and earlier peak in the TL curve compared to crackers alone. Such results suggest that hedonic enhancement in pairings may arise from the combination of contrasting and complementary attributes, with crackers balancing the stickiness and sweetness of the jam and the jam mitigating the dryness of the crackers. Although these interpretations remain qualitative, they provide a plausible link between ingredient properties, sensory attributes, and temporal liking outcomes. Future studies involving a wider variety of food categories and ingredient processing methods may further clarify how intrinsic product properties shape both TDS profiles and hedonic dynamics.

The emergence of wheat-like and dry attributes in crackers may be linked to structural protein elements (such as gluten network) and processing steps like baking that affect water content, crumb/crust structure, and flavor release in cereal products. Links between protein aggregation, thermal processing, and sensory attributes in flour-based goods are discussed in recent reviews and studies [51,52,53,54]. For example, it was demonstrated how increasing protein additions change dough viscoelasticity and affect hardness and mouthfeel in crackers [53]. However, a detailed discussion of ingredient composition or processing effects is beyond the scope of this study, which primarily aims to develop and evaluate predictive models of temporal liking from TDS data.

Table 4 presents the RMSEs of the 32 models examined. The ten models with the smallest (best) RMSEs and the ten with the largest (worst) RMSEs are listed in ascending order of RMSE. Ranks were determined based on the median RMSE values across the four food combinations, while the table also reports RMSEs for each individual combination.

The model performances varied substantially across the models. The top-ranked model achieved a mean RMSE of 0.44, with an IQR of 0.34–0.54. This model employed 128 neurons and a single auxiliary flag indicating whether the sample was a single food item or a cracker–spread pairing. In contrast, the lowest-ranked model, which used 64 neurons and five auxiliary flags, recorded a mean RMSE of 3.58.

## 5. Discussion

The median RMSE and IQR for the best-performing model were 0.44 and 0.34–0.54, respectively. Given that the liking score is based on a 9-point scale, this represents a small prediction error. In a previous study [25], the RMSE for predicting the liking of four types of coffee, when the model was trained only on data from the same brand, ranged from 0.46 to 0.59. In a study on strawberries [8], the RMSE ranged from 0.68 to 0.99. The top ten models used in the present study outperformed these previous results in terms of RMSE.

According to Table 4, the model with 128 neurons and a single auxiliary input flag exhibited the highest prediction accuracy. The top five models achieved comparable performance, with the number of auxiliary inputs ranging from 0 to 3. In contrast, models with four or more auxiliary input flags performed poorly, regardless of the number of neurons. A possible explanation is overfitting: when brand-level flags were provided, the model may have relied too heavily on this specific information, thereby improving the fit to the training data but diminishing generalization to unseen food items or pairings. By contrast, auxiliary inputs that only indicate broad categories (e.g., cracker, spread, or combination) appear to strike a better balance by providing useful context without overly constraining the model to brand-level information. These results suggest that the inclusion of brand flags negatively affected prediction accuracy, and that using at most three auxiliary input flags may be effective for improving estimation accuracy. However, since two of the top ten models did not utilize any auxiliary input flags, the overall effect of auxiliary inputs may be limited. While the findings indicate that employing a very small number of auxiliary input flags is likely beneficial, it would be premature to draw firm conclusions based solely on this limited set of food samples (crackers and spreads).

For problems of the scale addressed in this study, a reservoir size of approximately 128 to 192 neurons appears to be appropriate. In general, increasing the number of neurons improves the model’s capacity to address complex input–output relationships, but also increases the risk of overfitting [39,55]. Conversely, using too few neurons may result in an insufficient number of state variables to adequately explain the liking response. Although there are few studies investigating the relationship between problem or domain size and reservoir size for TL curve prediction, previous work has indicated a similar optimal range [8,25]. It should be noted, however, that only four neuron sizes (64, 128, 192, and 256) were tested in this study, and thus it cannot be excluded that optimal values might exist outside this range.

Considering these findings together, we were able to identify, to some extent, appropriate reservoir model configurations for food pairing prediction. The number of neurons should be in the range of approximately 128–196, and the use of auxiliary inputs, if any, should be limited to about one to three types. However, these characteristics may change if the number of food items to be processed increases.

Several approaches could be considered to further improve estimation accuracy. First, optimizing the number of participants is important. Although the optimal panel size for the TDS method itself has not been fully determined [56], the same holds true for the prediction of TL curves from TDS data. Additionally, it is not necessarily the case that binary flags are the only suitable auxiliary inputs; exploring flags with three levels or continuous values may also be beneficial. That said, as mentioned earlier, the top-performing models investigated in this study likely already achieve sufficiently high accuracy among approaches using reservoir computing. Looking ahead, it may be more important to devise strategies to prevent unexpectedly large estimation errors.

There are still some limitations and unresolved issues with the proposed method.

A limitation of this study is the restricted number of food items examined: two types of crackers and two types of spreads, yielding four pairings. While this limited scope constrains the generalizability of the findings to broader categories of food pairings, it was chosen as a practical compromise between experimental feasibility and participant burden. Expanding the number of food types would require alternative designs, such as incomplete block designs or reducing the number of pairings tested per participant. Future research should therefore investigate whether the proposed approach can be extended to a wider range of food items under such designs.

Another major limitation of the present approach concerns the normalization of time. The method assumes that all curves are temporally normalized. While this normalization increases the consistency of the analysis, it also results in the loss of information regarding individual differences, such as the rate and duration of flavor changes in the mouth. For example, the average tasting times for crackers and spreads were approximately 20 s and 10 s, respectively, but time normalization eliminates this information. At present, it is unclear how this normalization may have influenced the results.

We acknowledge that bootstrap resampling does not reproduce the true variability of an independent consumer population, as it relies on repeated sampling from the same limited panels. In this study, it was used solely as a data augmentation method to enable model training. Future work with larger and more diverse participant groups will be needed to validate the generalizability of the proposed approach.

In the current approach, training the model to predict the liking of food pairings requires data from all other pairings, which does not reduce the overall cost of sensory evaluation. Ideally, the model would generate the TL curve for a pairing solely from the TDS curves of the two individual foods. Furthermore, it would be advantageous if the training data consisted only of the TDS and TL curves for single items and a limited number of key pairings. For instance, with 10 types of crackers and 10 types of spreads, training the model using only the single-item data (20 foods) and a few critical pairings would substantially reduce the sensory evaluation workload. Although developing such a method presents considerable challenges, we aim to pursue this direction in future work.

While the present study does not directly quantify reductions in sensory evaluation costs, our approach is motivated by the industrial reality that sensory testing is widely conducted and often resource intensive. A recent survey [29] reported that sensory evaluations are used in nearly 70% of food development cases, frequently involving large consumer groups and expert panels, and that cost was among the most cited reasons for omitting expert evaluations. Against this backdrop, the present findings represent an initial methodological step toward predictive approaches that may, in the long term, contribute to reducing the burden of sensory evaluation in food development.

## 6. Conclusions

This study demonstrated the feasibility of predicting TL curves for food pairings based on TDS data using reservoir computing. By collecting TDS and TL data for two types of crackers, two types of spreads, and their combinations, we developed and evaluated several reservoir computing models with varying numbers of neurons and auxiliary input flags. The most accurate models achieved a median RMSE of 0.44 on a 9-point liking scale, which is a notably small prediction error when compared to previous research on temporal liking prediction. Models with a moderate number of neurons (128–192) and a limited number of auxiliary inputs (up to three) performed best, while models with excessive auxiliary input flags tended to perform poorly.

These results suggest that reservoir computing can provide robust predictions of hedonic dynamics in food pairings, with potential to reduce the scale and cost of sensory evaluations. Nevertheless, key limitations remain: the present models require data from all food pairings during training, which currently limits their practical benefit in reducing sensory evaluation workload. Furthermore, the use of temporal normalization, while enhancing analytical consistency, results in the loss of potentially informative dynamics.

Future research should address these limitations by developing methods that can generalize from data on single foods and a limited set of key pairings, ideally enabling the prediction of TL curves for any pairing from the TDS curves of its components. Despite these challenges, the present findings constitute an important step toward more efficient, data-driven approaches for understanding and predicting food-pairing preferences.

## Figures and Tables

**Table 1:** Food products.

Category	Name	Original Name	Manufacturer
Cracker	Plain	Premium Saltine Crackers	Nabisco, Inc.
	Sesame	Black Sesame Soft Crackers	Maeda Confectionery Co., Ltd.
Spread	Peanut	Verde Peanut Whip	Aohata Corporation
	Strawberry	Spoon Free Strawberry	Aohata Corporation

**Table 2:** Definitions of attributes used in the TDS tasks.

Attribute	Definition
Aromatic	Complex but pleasant fragrant
Burned	Well-baked, toasty
Buttery	Butter-like flavor
Dry	Moisture-free, crispy
Nutty	Reminiscent of dried nuts
Salty	Salty (basic taste)
Sesame-like	Sesame-like flavor
Smooth	Smooth mouthfeel, no graininess texture
Sour	Sour (basic taste)
Sweet	Sweet (basic taste)
Wet	Moist, no dryness
Wheat-like	Wheat-like flavor

**Table 3:** Auxiliary input dimensions and their descriptions.

Total Input Dimensions	Auxiliary Dimensions	Description
12	0	No auxiliary information (only TDS curves provided)
13	1	Combination flag (0: single item; 1: paired cracker and spread)
14	2	Cracker flag and spread flag (both set to 1 for paired samples)
15	3	Cracker, spread, and combination flags (exclusive, only one set to 1)
16	4	Brand flags (premium, sesame, peanut, strawberry)
17	5	Brand flags and combination flag
18	6	Brand flags, cracker flag, and spread flag
19	7	Brand flags, cracker, spread, and combination flags

**Table 4:** RMSEs for each model architecture. “Jam” refers to strawberry jam. Models are listed in ascending order of RMSE.

Rank	Neurons	Flags	Median RMSE (IQR)				
			**All Pairs**	**Plain–Jam**	**Plain–Peanut**	**Sesame–Jam**	**Sesame–Peanut**
1	128	1	0.44 (0.34–0.54)	0.41 (0.31–0.49)	0.46 (0.35–0.58)	0.42 (0.34–0.50)	0.51 (0.39–0.62)
2	192	2	0.44 (0.34–0.55)	0.39 (0.30–0.50)	0.42 (0.30–0.57)	0.42 (0.36–0.50)	0.52 (0.39–0.67)
3	192	0	0.46 (0.35–0.58)	0.42 (0.33–0.54)	0.40 (0.33–0.52)	0.45 (0.35–0.53)	0.59 (0.46–0.73)
4	64	1	0.46 (0.36–0.59)	0.44 (0.37–0.52)	0.48 (0.34–0.61)	0.42 (0.32–0.53)	0.59 (0.44–0.70)
5	192	3	0.46 (0.36–0.60)	0.46 (0.35–0.59)	0.49 (0.35–0.61)	0.41 (0.34–0.49)	0.57 (0.45–0.75)
6	256	1	0.47 (0.35–0.57)	0.44 (0.33–0.56)	0.46 (0.35–0.57)	0.45 (0.36–0.54)	0.51 (0.40–0.62)
7	192	1	0.47 (0.37–0.59)	0.44 (0.33–0.52)	0.47 (0.34–0.57)	0.46 (0.40–0.54)	0.57 (0.43–0.70)
8	256	0	0.47 (0.37–0.59)	0.44 (0.34–0.56)	0.50 (0.39–0.59)	0.43 (0.34–0.54)	0.50 (0.43–0.64)
9	128	2	0.48 (0.37–0.62)	0.60 (0.45–0.73)	0.47 (0.35–0.57)	0.41 (0.32–0.48)	0.52 (0.42–0.68)
10	256	3	0.48 (0.37–0.63)	0.41 (0.32–0.53)	0.44 (0.33–0.58)	0.47 (0.40–0.60)	0.62 (0.50–0.78)
⋮			⋮				
23	192	6	1.49 (1.13–1.71)	1.66 (1.46–1.83)	1.47 (1.39–1.61)	0.88 (0.71–0.99)	1.71 (1.56–1.89)
24	256	7	1.68 (1.05–3.76)	2.55 (2.30–2.83)	1.08 (0.86–1.26)	1.00 (0.85–1.22)	4.65 (4.47–4.90)
25	256	5	1.91 (1.08–2.25)	1.60 (1.40–1.87)	2.53 (2.34–2.75)	2.10 (1.95–2.21)	0.68 (0.56–0.86)
26	128	7	2.38 (0.82–4.72)	5.60 (5.31–5.86)	0.60 (0.44–0.79)	3.92 (3.73–4.12)	1.01 (0.89–1.23)
27	192	5	2.38 (1.65–4.08)	1.80 (1.61–1.97)	1.55 (1.43–1.67)	3.20 (3.01–3.38)	5.55 (5.41–5.68)
28	192	4	2.58 (1.14–4.53)	1.59 (1.42–1.77)	6.58 (6.34–6.77)	3.73 (3.56–3.88)	0.77 (0.60–0.96)
29	64	7	2.71 (2.05–3.72)	2.23 (2.04–2.49)	2.95 (2.83–3.11)	1.95 (1.73–2.08)	4.30 (4.12–4.47)
30	256	6	3.01 (2.00–4.74)	6.33 (6.16–6.52)	3.60 (3.37–3.81)	1.65 (1.40–1.93)	2.50 (2.24–2.68)
31	192	7	3.42 (1.61–5.25)	1.96 (1.62–2.20)	5.07 (4.89–5.21)	5.51 (5.30–5.70)	1.39 (1.23–1.56)
32	64	5	3.58 (2.28–4.14)	4.61 (4.44–4.79)	3.38 (3.22–3.57)	0.58 (0.44–0.74)	3.81 (3.63–3.95)

## Data Availability

The research data are available from the corresponding author upon direct request, accompanied by a clear explanation of the intended purpose.

## Abbreviations

The following abbreviations are used in this manuscript:

TDS	Temporal dominance of sensations
TL	Temporal liking
